# Underestimation of systolic pressure in cuff-based blood pressure measurement

**DOI:** 10.1093/pnasnexus/pgaf222

**Published:** 2025-08-12

**Authors:** Kate Bassil, Anurag Agarwal

**Affiliations:** Department of Engineering, University of Cambridge, Trumpington Street, Cambridge CB2 1PZ, United Kingdom; Department of Engineering, University of Cambridge, Trumpington Street, Cambridge CB2 1PZ, United Kingdom

**Keywords:** blood pressure, auscultatory method, systolic underestimation, measurement inaccuracy, arterial occlusion

## Abstract

High blood pressure (hypertension) is the number one risk factor for premature death. Hypertension is asymptomatic, so blood pressure must be regularly monitored to diagnose it. In auscultatory blood pressure measurement, a patient’s systolic (maximum) and diastolic (minimum) blood pressure are inferred from the pressure in an inflatable cuff wrapped around the arm. This technique is the gold standard against which all other noninvasive devices are validated. However, auscultatory measurements systematically underestimate systolic blood pressure and overestimate diastolic blood pressure. Overestimation is attributed to the increased cuff pressure needed to occlude the artery because of the surrounding tissue and arterial stiffness. In contrast, the cause of systolic underestimation, which leads to potentially a third of systolic hypertension cases being missed, has remained unclear. When the cuff is inflated beyond the systolic blood pressure, the blood flow to the vessels downstream of the cuff is cut off. The pressure in these downstream vessels drops to a low plateau. We have developed a novel experimental rig that shows that the low downstream pressure is the key cause of the underestimation of systolic blood pressure. The lower the downstream pressure, the greater the underestimation. Our results yield a simple physical model for the underestimation of systolic pressure in our rig and in the human body. Understanding the physics behind the underestimation of systolic blood pressure paves the way for developing strategies to mitigate this error.

Significance Statement The cuff-based auscultatory method is the gold standard for blood pressure measurement, but it remains inaccurate. While the overestimation of diastolic (minimum) blood pressure is well understood, the consistent underestimation of systolic (maximum) blood pressure remains unexplained. Previous experimental setups have neither observed nor investigated this underestimation. With a novel experimental model, we provide an explanation for the underestimation of systolic blood pressure and a better understanding of the physics behind auscultatory measurement. Our findings provide a foundation for developing improved calibration methods or refining the auscultatory measurement protocol, offering a pathway to enhance the accuracy of noninvasive blood pressure measurements and reduce diagnostic errors.

## Introduction

The measurement we refer to as “blood pressure” is not a constant value but oscillates between a maximum (systolic) and minimum (diastolic) value with each contraction and relaxation of the heart (a cardiac cycle). Systolic and diastolic blood pressure are critical measures of human health. Hypertension, the condition of persistently raised blood pressure, is the number one risk factor for premature death worldwide (1, 2). It is also typically asymptomatic, so it is unlikely to be apparent until the resulting health problems become significant. Early identification of an asymptomatic condition requires regular and accurate testing.

For regular testing across a large portion of the population, low-cost, widely accessible, and noninvasive testing methods are essential. Direct blood pressure measurement with an arterial line can be accurate but is expensive and invasive (3). Indirect measurement techniques are therefore used for routine blood pressure testing. The gold standard amongst noninvasive blood pressure measurement techniques, against which other devices are validated, is cuff-based auscultatory measurement. For example, new noninvasive measurement devices should give mean results within 5 mmHg of the auscultatory readings for both systolic and diastolic pressures (4). Because of these validation requirements, improvements in auscultatory measurement will translate to greater accuracy in other measurement techniques, such as the widely used automatic, oscillometric blood pressure monitors.

### The auscultatory method

**Fig. 1.**
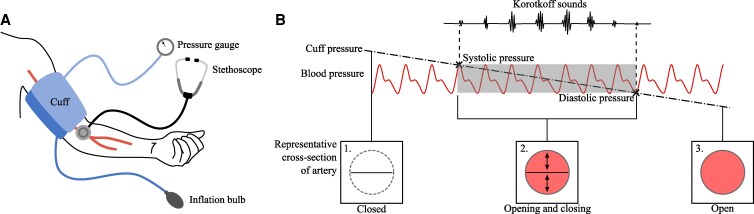
A) Setup for auscultatory blood pressure measurement. B) Representative artery behavior during auscultatory measurement.

In the auscultatory method, an inflatable cuff is placed around the upper arm, as shown in Fig. 1A. The cuff is inflated to a pressure above the systolic pressure. The pressure in the cuff is then gradually released at 2–3 mmHg/s (5). At a critical cuff pressure, periodic tapping sounds known as Korotkoff sounds start, and can be heard through a stethoscope. This critical pressure is recorded as the systolic blood pressure (SBP). As the pressure is reduced below the SBP, Korotkoff sounds are continuously heard until a second critical pressure is reached, when these sounds stop. This cuff pressure is recorded as the diastolic blood pressure (DBP).

Figure 1B illustrates the relationship between the cuff pressure, blood pressure, artery cross-section, and Korotkoff sounds. With the cuff pressure initially raised above the systolic pressure, the artery is occluded (6) and remains fully closed throughout the cardiac cycle, as shown in cross-section 1 of Fig. 1B. The cuff pressure is then gradually decreased and drops below the SBP. In the range SBP > cuff pressure > DBP, shaded in gray in Fig. 1B, the pressure inside the artery is higher than the applied cuff pressure during a portion of each cardiac cycle. For this period of the cycle, the artery can open, and blood can flow through it. For the remainder of the cycle, when the blood pressure drops back below the cuff pressure, the artery is closed. Initially, when the cuff pressure is only slightly below the SBP, the artery opens only for a brief portion of each cardiac cycle. As the cuff pressure decreases further, the artery remains open for a progressively larger portion of each cycle. When the cuff pressure falls below the DBP, the external pressure no longer surpasses the internal pressure at any point in the cycle, allowing the artery to remain fully open, as shown in cross-section 3 of Fig. 1B. The opening and closing of the artery in the range SBP > cuff pressure > DBP produces Korotkoff sounds^a^ (7, 10, 13, 14). Outside of this range, the artery is either fully closed or fully open, and there is silence (14).

### Inaccuracy of cuff-based measurement

The description above is an idealized version of auscultatory measurement. If the artery closed as soon as the cuff pressure exceeded the internal pressure, as described, then the measured cuff pressure would be a perfect proxy for the arterial blood pressure.

However, in practice, the auscultatory method is inaccurate, systematically underestimating systolic pressure and overestimating diastolic pressure. Meta-analysis of 74 studies by Picone et al. (15) shows that cuff-based blood pressure measurements underestimate SBP by an average of 5.7 mmHg and overestimate DBP values by an average of 5.5 mmHg. The diagnostic impact of these errors is substantial: Turner et al. (16) found that systematically overestimating DBP by 5 mmHg increases the number of patients with a measured DBP over 90 mmHg, the threshold for hypertension, by 132%. Equivalent underestimation of SBP results in 30% of patients with systolic hypertension (SBP >140 mmHg) being missed.

The physical factors contributing to overestimation are well established. Firstly, the cuff pressure is not applied directly to the artery, but to the surface of the arm. The arm tissue reduces the pressure transmitted to the artery, so the external load experienced by the artery is lower than the cuff pressure (17–19). Secondly, additional pressure is required to overcome the buckling stiffness of the artery wall. Therefore, the artery will only close once the external pressure exceeds the internal pressure by a certain threshold, known as the buckling pressure (20). These factors cause the measured cuff pressure to overestimate the blood pressure (17, 21).

Given these existing explanations for inaccuracy, it might be expected that the auscultatory method would overestimate both SBP and DBP. The surrounding tissue and artery stiffness are predicted to increase the cuff pressure required to keep the artery closed and so to cause the artery to reopen early during cuff deflation, while cuff pressure still exceeds SBP. However, intra-arterial pressure measurements taken by Celler et al. (12) during cuff deflation show that, rather than the artery opening early, the reopening of the artery is substantially delayed. In these measurements, the brachial artery remains closed down to cuff pressures as much as 24 mmHg below the systolic pressure (12). There is currently no physical explanation for this delayed opening and the underestimation of SBP it produces. This work aims to identify the cause of underestimation of SBP in auscultatory measurement, addressing a critical gap in our understanding of cuff-based blood pressure measurement.

### Underestimation of systolic pressure

**Fig. 2.**
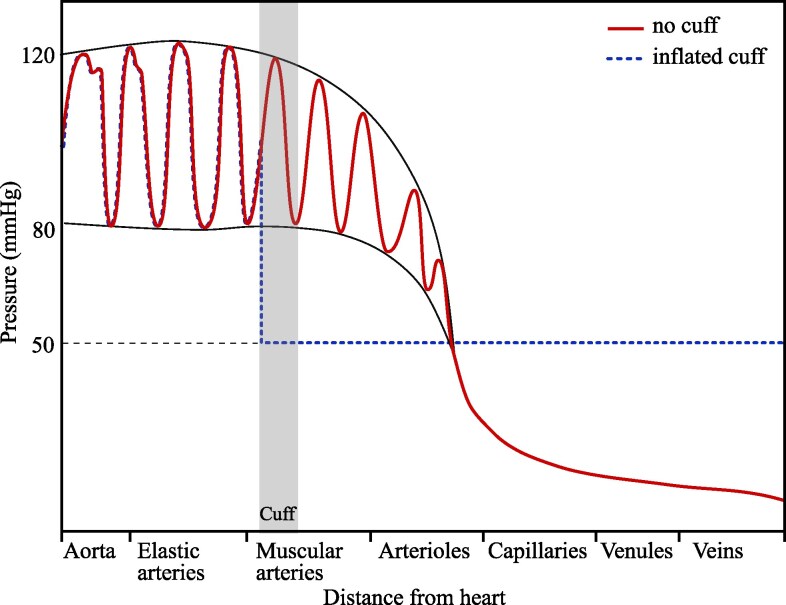
Variation of pressure throughout the blood vessels. The solid red trace shows pressure variation when the vessels are undisturbed, with no cuff applied (adapted from Ref. (22). Access for free at https://openstax.org/books/anatomy-and-physiology/pages/1-introduction). The envelope around the trace shows the SBP and DBP. The dotted blue trace shows the blood pressure variation that occurs when the brachial artery is closed by an inflatable cuff around the upper arm. This plot shows the steady state condition once the pressure has equalized in the downstream vessels (on initial closure, blood will flow from the higher pressure arteries to the lower pressure veins until the pressures level out (23)).

To explain underestimation in auscultatory measurement, we propose a new model that takes a more global view of the circulatory network, accounting for the variation of blood pressure along the length of the artery. A schematic of the pressure variation throughout the circulatory system is shown in Fig. 2 (adapted from Ref. (22)). The variation in blood pressure when the arm is undisturbed, before the blood pressure cuff is applied, is shown in solid red. The pressure decreases with distance from the heart (22), continuing to drop in the smaller arteries, capillaries and veins, where the pressure approaches 0 mmHg.

When an inflated cuff occludes the artery, the blood flow to the vessels distal to the cuff (downstream of the point of closure) is cut off. In the absence of flow, the blood in the downstream network of vessels becomes static and the pressure in the vessels reaches an equilibrium. This means that the arterial pressure directly distal to the cuff drops towards the pressure of the rest of the downstream vessels, eventually plateauing at a low, constant pressure usually between 30 and 70 mmHg (6). With the downstream vessels separated from the heart, there are no pressure oscillations (6, 12, 23–25). This behavior is shown in dotted blue in Fig. 2. The pressure on the proximal side of the cuff, the side closer to the heart, is unaffected (24), while the pressure on the distal side drops to a constant value, which is 50 mmHg in our example. This low plateau will be present when the SBP is recorded because the cuff is inflated at the start of the auscultatory measurement, so the artery is closed. Regression analysis of in vivo measurements by Celler et al. (26) showed that the difference between the pressure downstream of the cuff and the systolic pressure accounted for almost 43% of the variance in systolic measurement error in a cohort of 40 subjects. However, the physics behind this correlation has not been investigated. In parallel with these in vivo measurements, initial tests in our laboratory rig (27) suggested a relationship between downstream pressure and underestimation. Together, these findings motivate the study presented in this article to establish a causal link between the lowered downstream pressure and the underestimation of SBP. We have achieved this through controlled, independent variation of the downstream pressure in our laboratory rig, which cannot be produced in vivo. Previous lab-based investigations (28–30) did not observe the underestimation of SBP, because their setup did not reproduce the total closure of the artery and the resulting drop in pressure downstream of the cuff.

## Results and discussion

Reproducing the auscultatory technique in an experimental rig allows us to isolate and measure many variables inaccessible in the body, including upstream and downstream pressures. This controlled environment enables us to systematically and repeatably alter these variables, simplifying the complex in vivo physiology to focus on the physics involved in systolic blood pressure measurement. We designed the setup to capture the essential physics of underestimation, but it differs from in vivo conditions in aspects such as the material and dimensions of the artery model, and the absolute pressures. Consequently, we avoid direct numerical comparisons with in vivo data, instead emphasizing the trends in behavior and the causal relationships observed in our experiments.

### Experimental rig

**Fig. 3.**
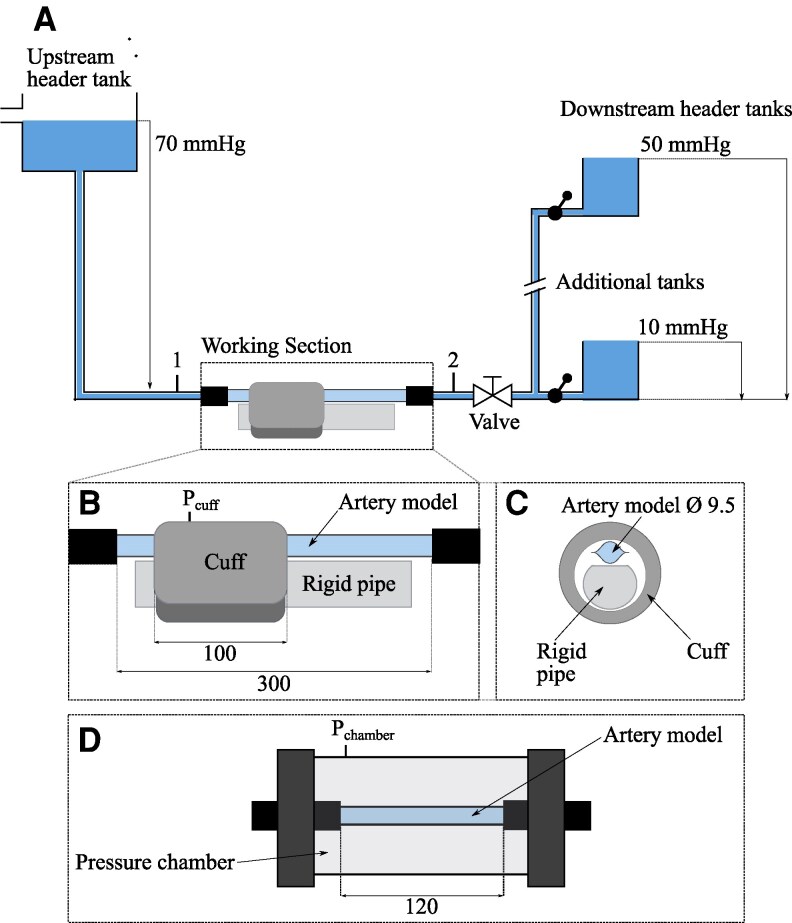
A) Schematic of the experimental rig, with upstream and downstream header tanks setting the pressure conditions on either side of the Working section. The downstream pressure is varied by switching between downstream header tank heights. B) Details of the Working section of the rig, where the artery model is compressed by a small blood pressure cuff (Omron CD-CS9). C) Cross-section of Working section. D) An alternative Working section, where the external load is applied to the artery model with a pressure chamber instead of a blood pressure cuff, to provide uniform loading. All lengths in mm.

**Fig. 4.**
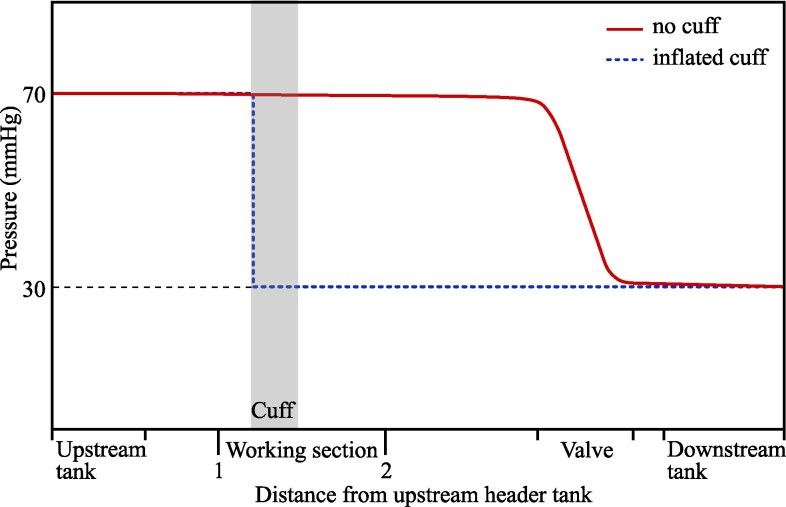
Variation in pressure throughout the experimental setup. Labels on the “Distance from upstream header tank” axis correspond to the same labels on the rig schematic in Fig. 3.

A schematic of the in vitro rig is shown in Fig. 3. Water is used as the working fluid throughout these experiments. It captures the core dynamics of blood flow and pressure, particularly the relationship between downstream pressure and arterial closure, while simplifying the experimental setup. Previous studies have shown that the mechanics of auscultatory measurement are consistent across a wide range of viscosities, including water and blood (28) (see Supplementary material: “Using water as the working fluid”). The water flows from the upstream header tank through the artery model in the “Working section” (labelled in Fig. 3) and into one of the downstream tanks, which set the downstream pressure conditions and are interchanged between tests. The pressure variation throughout the rig is shown in Fig. 4. As in Fig. 2, the solid red trace shows the pressure variation with no cuff, while the dotted blue shows the pressure with the cuff inflated.

The use of a constant pressure header tank means that the “blood pressure” in the rig is not oscillatory. This results in a single opening of the artery during cuff deflation rather than the repeated opening and closing observed in vivo. When this single reopening occurs, the downstream pressure rises from a low plateau, reproducing the behavior in the body at the point of systolic measurement, when the first opening of the artery occurs. With a constant upstream pressure, we reproduce the closure and reopening of the artery and are able to isolate the effect of downstream pressure on this reopening while avoiding the added complexity of oscillatory flows. The upstream header tank maintains a fixed pressure of 70 mmHg at location 1 ($P_{1}$), upstream of the cuff, irrespective of the flow resistance. Therefore, the pressure does not rise when the cuff is inflated and the artery model closes. This condition replicates the response in the body, where arterial line measurements show no change in upstream blood pressure in response to the closure of the downstream arteries with a cuff (24). An alternative approach, used in previous experiments (28–30), employs a pump to produce oscillatory flow, with a parallel route to bypass the closed artery. However, with this setup, the upstream pressure will still vary when the artery opens and closes due to the change in the total network resistance. As we are specifically interested in the mechanics and pressures at the point of opening, when this change in resistance would occur, we must avoid introducing even these small variations. Additionally, the parallel route prevents the isolation of the downstream section, making it difficult to achieve the low downstream pressure plateau required for our investigation. To avoid these issues, we simplify the flow system by using a steady upstream pressure.

The low pressure distal to the cuff when the artery model is closed (in dashed blue in Fig. 4) is possible because, unlike previous setups (28–30) the flow network is not a closed loop, so the inlet and outlet conditions can be set independently. The downstream pressure, at location 2 ($P_{2}$), is controlled by varying the height of the tanks at the outlet to give pressures between 10 and 50 mmHg. When the artery model in the Working section is open (in solid red in Fig. 4), $P_{2}\approx P_{1}$, with only a small drop in pressure along the artery due to the frictional resistance to the flow. This is a good match to the in vivo behavior, where the average arterial pressure is close to constant along the upper arm (see Fig. 2). There is then a large pressure drop between location 2 and the downstream tank, which is produced with an adjustable valve. In vivo, this pressure drop would happen gradually along the remaining downstream vessels, as shown in Fig. 2. The role of the valve in the rig is to ensure that, when the artery is open, the majority of the pressure drop occurs across the valve, rather than across the artery model. When the artery model is closed, there is no flow and the downstream section is isolated from the upstream header tank. The pressure in the whole section distal to the cuff (measured at location 2) drops to that of the downstream tank, as shown in dashed blue in Fig. 4. The open and closed conditions in the rig replicate those in the body, but with an absence of oscillations in the rig. Although the pressure differences in our rig, of 20–60 mHg, are smaller than that observed in vivo, of around 50–90 mmHg for a healthy person (6), they are large enough to reproduce the physical effect of the pressure drop in the body.

In the primary Working section (Fig. 3B and C), the tube representing the artery is compressed against a rigid pipe under an inflatable blood pressure cuff. The artery model is in direct contact with the cuff, with no surrounding tissue. There is no standard approach to experimentally modeling the surrounding tissue in auscultatory measurement and the loading of the artery is often analyzed in isolation (29, 31). Potential tissue models such as an air and water filled tube (32), or a homogenous, low stiffness silicone (19) do not produce realistic loading conditions. The air and water filled tube applies uniform pressure to the artery, which differs from the uneven pressure distribution observed in vivo (18), while a silicone surrounding significantly increases the cuff pressure required for buckling (by ∼70 mmHg in (19)). Given these limitations, we chose to exclude surrounding tissue and focus on the closure and reopening of the artery in isolation, without the confounding effects of unrealistic tissue models.

Previous studies have used rubber models for the artery (28–30). Rubber tubes are commonly used to investigate the effect of buckling stiffness on overestimation. However, these cylindrical tubes are a poor replica of arteries under large external pressures. They rapidly increase in stiffness as their cross-sectional area approaches zero and remain partially open at transmural pressures of up to 40 mmHg (33). In contrast, complete closure of the artery is observed in the body with only a small transmural pressure, close to zero (33). With the incomplete closure of rubber tubes, the absence of flow and the resulting low downstream pressure observed in vivo cannot be recreated.

To overcome this issue, we use lay-flat tubes to model the artery. While lay-flat tubes differ in form from the brachial artery—with a noncircular shape, low buckling stiffness, and minimal elasticity—they exhibit a critical feature missing from previous experimental setups: low resistance to collapse, down to a zero cross-sectional area (32). This allows for complete closure of the tube and so produces the downstream pressure plateau observed in vivo. Thus, lay-flat tubes are essential for our investigation, despite their differences from real arteries.

The pressure applied by an inflatable cuff varies along the length of the cuff (18). To study if this impacts blood pressure measurement, we conducted a series of tests using an air pressure chamber to apply the external pressure instead of a blood pressure cuff. Pressure applied in this way is entirely uniform. This alternative Working section is shown in Fig. 3D. Both the cuff and the pressure chamber are inflated and deflated manually.

### Measured cuff pressure

**Fig. 5.**
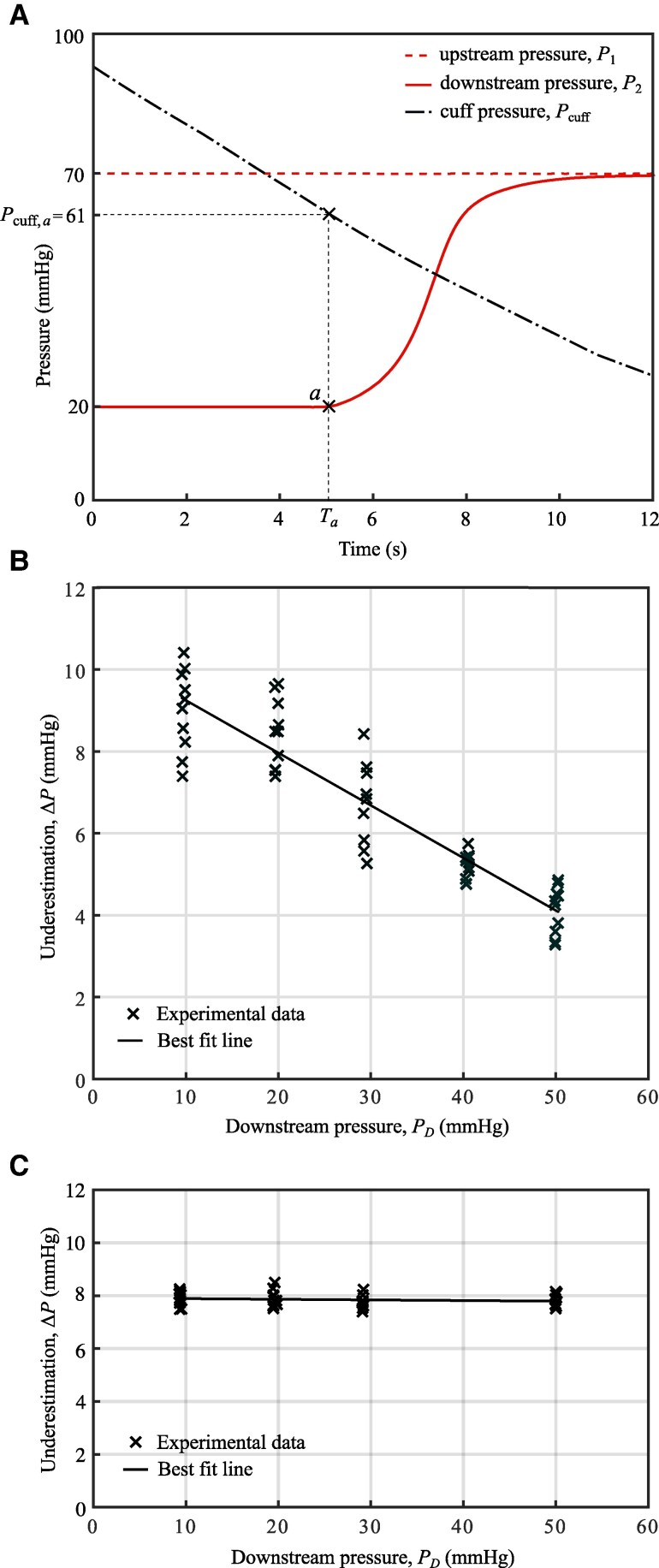
A) Pressure traces from a single test run, with a downstream pressure of 20 mmHg. Measuring the cuff pressure at the time of reopening, $T_{a}$, gives the reading $P_{\mathrm{cuff},a}=61$ mmHg, a 9 mmHg underestimation of the upstream pressure. B) Underestimation of the upstream pressure, ΔP, measured using a manual blood pressure cuff, for downstream pressures 10–50 mmHg. The equation of the best fit line is $\Delta P=-0.13P_{D}+10.53$. C) Underestimation of the upstream pressure measured using a pressure chamber. The equation of the best fit line is $\Delta P=-0.0024P_{D}+7.91$. The underestimation is constant across the different downstream pressures tested.

Figure 5A depicts an example test run. The cuff is initially inflated to a pressure greater than the upstream pressure and, because the flow through the artery is cut off, the downstream pressure, $P_{2}$, drops to that at the downstream tank (20 mmHg in this case). The upstream pressure, $P_{1}$, remains constant at 70 mmHg, set by the upstream header tank. The cuff around the model artery is then gradually deflated. The rise in the downstream pressure at time $T_{a}$ indicates the opening of the model artery. (Details of selecting the threshold pressure for opening are provided in the Materials and methods section.) The cuff pressure at this time is recorded ($P_{\mathrm{cuff},a}$). In vivo, this first opening would produce a Korotkoff sound (26), and the cuff pressure would be recorded as the “measured SBP.” In the rig, the cuff pressure is recorded as the “measured upstream pressure,” where the upstream pressure represents systolic pressure. The underestimation of the upstream pressure is the difference between the true upstream pressure, $P_{1}=70$ mmHg and the measured upstream pressure, $P_{\mathrm{cuff},a}=61$ mmHg. So, for this test, the underestimation of the upstream pressure is ΔP=9 mmHg. The test is repeated with different downstream pressures, set by varying the height of the outlet tanks. The underestimation of the upstream pressure is plotted against these downstream pressures in Fig. 5B. There is a clear negative correlation between the downstream pressure and the underestimation of the upstream pressure, with R=−0.92 ($p=1.7\times 10^{-20}$). Lowering the pressure downstream of the cuff increases the underestimation of the upstream pressure.

### Loading with pressure chamber

Figure 5C shows the results obtained using the pressure chamber to apply the external loading to the artery model instead of the cuff. Unlike in the cuff case, the downstream pressure does not affect the underestimation of the upstream pressure.

### Sources of variability

In Fig. 5B, alongside the strong negative correlation between downstream pressure and underestimation, a noticeable scatter can be observed in the recorded underestimation at a fixed downstream pressure. This scatter arises primarily from variability in the loading applied by the blood pressure cuff. Unlike the pressure chamber case (Fig. 5C), where loading is uniform and highly repeatable, the cuff introduces variability due to slight differences in its position and pressure distribution between tests. This variability is exacerbated by the absence of surrounding tissue, which in vivo would dampen some of the loading variations. Additionally, the lay-flat tube models contribute to the variability, as they tend to wrinkle unpredictably during closure. Combined with the variation in cuff loading, this wrinkling reduces the reproducibility of tube closure and reopening, leading to the scatter observed in Fig. 5B. This scatter is more pronounced at lower downstream pressures, where the tube collapses more unevenly on the downstream side. Future studies could explore the use of alternative tissue and artery-mimicking materials to better replicate the in vivo environment and reduce this variability.

### Causal mechanism for underestimation

We have established that low downstream pressure results in the artery model reopening at a lower cuff pressure. We propose the following explanation for this relationship.

**Fig. 6.**
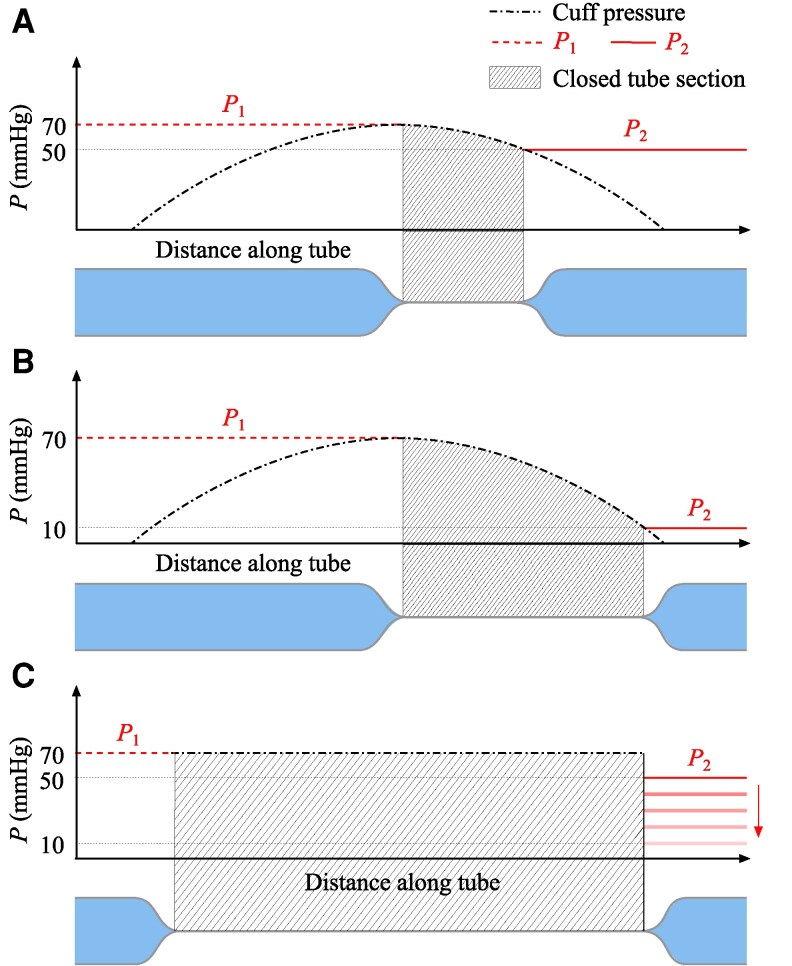
Each graph shows the internal pressure (P_1_ and P_2_) and external pressure (Cuff or chamber pressure) along the length of the tube. Below each graph is a schematic of the tube, with the closed section of the tube indicated by hatched lines. The length of tube closure depends on the downstream internal pressure, $P_{2}$ (shown in solid red), while the upstream pressure, $P_{1}$, is held constant at 70 mmHg (shown in dashed red). A) Downstream pressure of 50 mmHg, with external pressure applied by a cuff reading 70 mmHg. B) Downstream pressure of 10 mmHg, with external pressure applied by a cuff reading 70 mmHg. The length of tube closure increases with decreased downstream pressure. C) Variable downstream pressure (50–10 mmHg), with the cuff replaced by an air pressure chamber that applies a constant external pressure of 70 mmHg. The length of closure remains constant, irrespective of the downstream pressure.

Figure 6A and B shows the artery model, hereafter referred to as the tube, compressed by a cuff. The applied cuff pressure of 70 mmHg is the same in each case, while the downstream pressures are 50 and 10 mmHg, respectively. The pressure applied by the cuff is illustrated in dot-dashed black. Although the cuff pressure reading is 70 mmHg, the applied pressure will not be constant along the length of the cuff (17–19). The pressure is highest at the center, with lower pressure towards the edges of the cuff. This means the external pressure applied to the tube exceeds the internal pressure only over a fraction of the cuff length. Therefore, when the tube section central to the cuff is closed, the tube is still open on either side of the center. The length of the closed section depends on the internal pressure on either side of the point of closure (P_1_ and P_2_). In Fig. 6A, with an upstream pressure of 70 mmHg and a downstream pressure of 50 mmHg, the tube is open on the proximal (upstream) side and closes towards the center, where the external pressure reaches 70 mmHg. The tube is then closed until the external pressure is below 50 mmHg, further down the tube. The internal pressure exceeds the external pressure for the remaining length, so the tube is open. In Fig. 6B, the downstream pressure is decreased to 10 mmHg. The proximal side of the tube remains open as before, but a longer section is closed, as the tube only reopens where the external pressure drops below 10 mmHg.

When the cuff is deflated, the tube reopens from the upstream side as the pressure wave travels along the tube. With a greater length of the tube closed, the tube takes longer to fully reopen, so the opening time, $T_{a}$ from Fig. 5A, is later. Therefore, $P_{\mathrm{cuff},a}$ will reach a lower value by this time, resulting in a greater underestimation of the upstream pressure, ΔP. The lower the downstream pressure, the greater the closure length and, therefore, the larger the underestimation.

The results from the pressure chamber tests strongly support this theory. The applied external pressure is uniform across the tube length in the pressure chamber case, as shown in dot-dashed black in Fig. 6C. There is no decrease in external pressure away from the center. Therefore, when the measured external pressure exceeds the upstream pressure, the entire length of the loaded tube closes, irrespective of the chosen downstream pressure. Unlike the cuff, the pressure chamber is transparent, so the total closure of the tube can be observed in each test, over the full range of downstream pressures, from 10–50 mmHg. The upstream pressure will still be underestimated because of the time required for the pressure wave to travel along the tube. However, the downstream pressure will not affect this underestimation, since the length of the tube closure is independent of the downstream pressure.

### Application to in vivo measurement

With our experimental setup, using constant upstream pressure, we have determined that the length of artery closure under an inflated cuff depends on the downstream pressure. In the in vivo case, the key difference is the presence of oscillatory pressure upstream of the artery closure. However, the downstream pressure still drops to a low plateau upon artery closure, and, as in our experimental setup, the length of artery closure in vivo will depend on this downstream pressure. The artery will close over the length where the cuff pressure exceeds the downstream pressure, as in Fig. 6. In vivo, when the cuff is inflated above the SBP, the downstream pressure falls to a plateau of 30–70 mmHg (6) compared with a healthy systolic pressure of 120 mmHg. With a standard 12 cm cuff, the length of artery closure under the distal half of the cuff will vary between 0 and 6 cm, depending on this downstream pressure. The presence of oscillations from the cardiac cycle changes the details of how the length of artery closure affects the level of underestimation, but the principle remains similar—longer closure length means a greater time for reopening.

The pressure pulses produced by the heart propagate as waves along the arteries (34) at the “pulse wave velocity.” With the cuff pressure just below the systolic pressure, the artery will be closed for most of each cardiac cycle. When a systolic pressure peak arrives at the proximal side of the cuff, the artery will start to open as the blood pressure pulse travels along the artery under the cuff. But, the time for which the blood pressure exceeds the cuff pressure, and the pulse can therefore propagate under the cuff, is very small. This time may not be long enough for the pulse to reach the distal edge of the cuff. For example, let us consider a closure of 6 cm. The pulse wave velocity is much lower under the cuff than in the uncompressed artery, with values of ∼1 m/s under the cuff (10) compared to typical values of 5 to 20 m/s in the uncompressed artery (35–37). With this low pulse wave velocity of 1 m/s under the cuff, the rising front of the pulse wave would take 0.06 s to travel from the proximal side of the closed length to the distal side. For the pulse wave to successfully propagate to the distal side of the cuff, the blood pressure must exceed the cuff pressure for at least this duration of 0.06 s. Otherwise, the blood pressure at the proximal side of the cuff will drop back below the cuff pressure, and the artery will close before the wavefront can reach the distal edge of the cuff. The blood pressure pulse will be unable to propagate, so no Korotkoff sound will be heard.

**Fig. 7.**
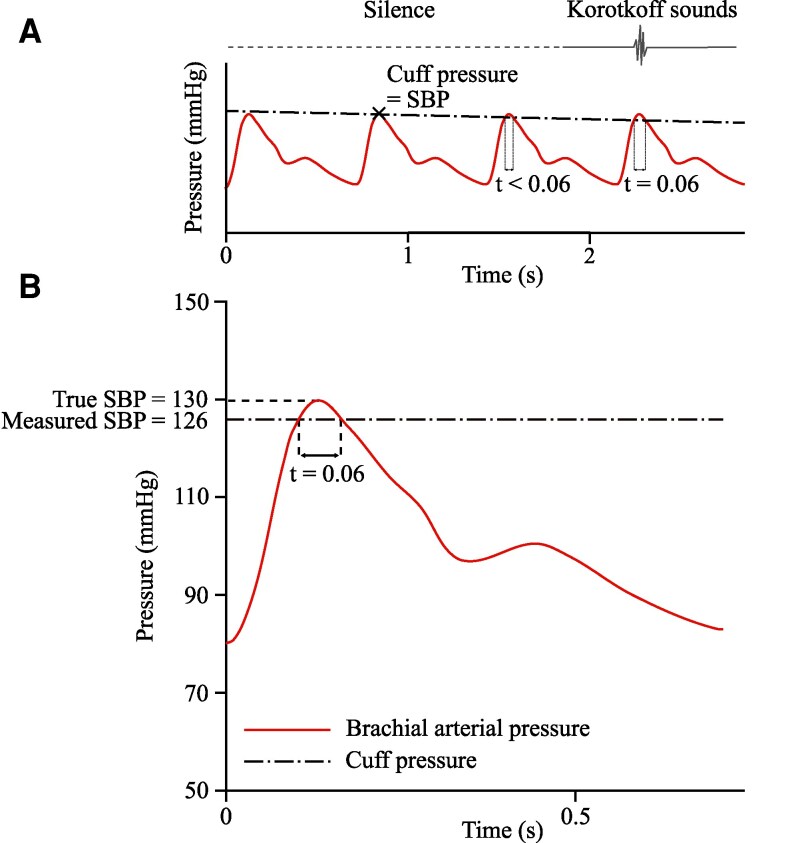
An illustration of the underestimation resulting from 6 cm of closure. A) Cuff pressure and arterial blood pressure as the cuff is deflated during an auscultatory measurement. No sounds are heard until the blood pressure exceeds the cuff pressure for 0.06 s. B) For a typical arterial waveform (adapted from Ref. (38)), the cuff pressure must drop to 126 mmHg to achieve the 0.06 s required for the artery to open, leading to a 4 mmHg underestimation.

Figure 7 illustrates the level of underestimation that could be caused by 6 cm of artery closure. Figure 7A shows representative cuff pressure and arterial blood pressure traces as the cuff is deflated during an auscultatory measurement. No Korotkoff sounds are heard until the blood pressure exceeds the cuff pressure for 0.06 s. This does not occur until the fourth blood pressure pulse, despite the cuff pressure equaling the SBP by the second pulse. In Fig. 7B, we evaluate the underestimation that results from this required 0.06 s for opening. For this standard waveform, reproduced from brachial artery measurements by Ref. (38), the SBP will be measured as 126 mmHg, while the true SBP is 130 mmHg; a 4 mmHg underestimation. The mechanics resulting in underestimation are demonstrated here for one specific closure length, and one exemplar pressure waveform. The level of underestimation depends on the shape of the blood pressure pulse, the pulse wave velocity and the length of closure of the artery, which is governed by the downstream pressure.

Note that the downstream pressure is low only at the start of the auscultatory measurement when the cuff has been inflated to above the SBP, completely occluding the brachial artery. When the DBP is recorded based on the cessation of the Korotkoff sounds, the artery is opening and closing with each cardiac cycle. The distal vessels are, therefore, not isolated from the upstream pressure pulses, and so the downstream pressure is not low (12). This cause of underestimation would, therefore, only affect the systolic measurement, in line with the meta-analyses (15, 39).

### Effect of deflation rate

In the rig, the deflation rate of the cuff (or pressure chamber) will influence the underestimation. A more rapid deflation causes the cuff pressure to decrease further in the time required for the tube to fully reopen (see Fig. S4). To reduce the effect of the deflation rate on our results, for the data shown in Fig. 5 we carried out the tests over a narrow range of deflation rates. The influence of the deflation rate on the underestimation data in Fig. 5b is shown in Fig. S5. The small variation in deflation rate across these tests has no significant impact on the underestimation.

The influence of the deflation rate is different in the oscillatory case to the constant upstream pressure case from the rig. The time for the artery to open in vivo is of the order of 0.1 s (10), compared with the order of seconds in the rig. Therefore, even for high deflation rates, there will be very little drop in cuff pressure in the time taken for the artery to open, so the deflation rate will not significantly affect the underestimation caused by the low downstream pressure. However, the deflation rate is still important as it affects the measurement accuracy more generally (40, 41). This is why a low deflation rate of 2–3 mmHg/s is recommended for in vivo tests (5). Details of the effect of deflation rate in vivo are provided in the Supplementary material: “A note on deflation rate.”

### Conclusions

We show that, to replicate the underestimation of the systolic blood pressure in a rig, it is essential for the model artery to close completely when the cuff pressure exceeds the systolic pressure, as observed in the body. The closure shuts off the blood supply to the vessels distal to the cuff, causing the pressure in the downstream section of the brachial artery to drop significantly. In our rig, we demonstrate that this pressure drop results in an underestimation of the systolic, but not the diastolic, blood pressure. The lower the downstream pressure, the greater the underestimation of the systolic blood pressure.

Because of the variable pressure applied by the cuff along its length, lowering the downstream pressure results in a greater length of artery closure. A longer closure length, in turn, means more time is required for the artery to open, both in the rig and in vivo, producing greater underestimation. This theory is supported by the comparison between cuff and pressure chamber results and could be further validated with in vivo ultrasound measurements using a methodology similar to that presented in Ref. (10).

In this article, we have identified the cause of the previously unexplained underestimation of systolic blood pressure. With this new understanding of the underlying mechanics of auscultatory measurement, we are now in a position to improve the measurement technique at both a device and a procedural level. For device manufacturers, engineering innovations such as cuffs with improved pressure uniformity could directly address the influence of downstream pressure. Clinical procedural changes could also be implemented with existing devices. For example, elevating the arm prior to the measurement, to reduce venous pressure, could produce predictable downstream pressure levels and hence predictable underestimation, which could then be accounted for in the reported reading. The underestimation could also be corrected for using patient-specific physiological parameters that correlate to downstream pressure. These parameters should be identified through in vivo testing and may include age, arm circumference, and pulse wave velocity. Calibration factors can either be applied by clinicians or incorporated by manufacturers into next-generation firmware for real time measurement adjustment. These approaches will tackle a critical source of diagnostic inaccuracy, improving not only the accuracy of auscultatory measurement itself but that of new oscillometric and cuffless blood pressure measurement devices, which are validated against manual auscultatory measurements.

Translating our new understanding of the physics behind underestimation into practice will require coordinated efforts across clinical and engineering disciplines. Clinical trials are needed to validate correction factors and procedural modifications, which could then be implemented by manufacturers and clinicians. By applying fundamental biomechanics to real-world applications, this work lays the groundwork for more accurate hypertension detection and better cardiovascular outcomes at scale.

## Materials and methods

### Fabrication of lay-flat tubes

Lay-flat tubes of width 15 mm and length 300 mm were produced by sealing the edges of 440 gauge polythene film (Transpack) on a strip heater.

### Data acquisition and processing

Pressure sensors (24PCCFA2G, Honeywell) were used to measure the water pressure at locations 1 and 2 in Fig. 3. The cuff (and chamber) pressure was measured with a Kulite pressure transducer (XCS–093–5PSID). The pressure measurements were taken simultaneously, at a frequency of 2 kHz, using a NI PXIe–1082 chassis, with a PXIe–4499 analog input module. The data were postprocessed in MATLAB, where calibration factors were applied. The data were digitally filtered using a fourth-order low-pass Butterworth filter with a cut-off frequency of 10 Hz. This cut-off frequency was chosen to remove noise which could trigger the opening threshold early.

### Calibration

The pressure sensors were calibrated using a mercury manometer. The calibration curves and equations are given in Figs. S1 and S2.

### Defining the threshold for opening

In the rig, the opening of the tube is defined by the rising of the downstream pressure from a low plateau, rather than from the presence of Korotkoff sounds. Therefore, there is no discrete value to identify it. Instead, the downstream pressure rises continuously and a threshold value for opening is required. Threshold pressures of 0.03, 0.2, 0.5, and 1.0 mmHg were tested. The observed relationship between downstream pressure and underestimation of upstream pressure is present in all four cases, with little change in the gradient of the graph of underestimation against downstream pressure (see Fig. S3). A threshold of 0.2 mmHg was used throughout.

## Supplementary Material

pgaf222_Supplementary_Data

## Data Availability

Additional data related to this publication are available at Apollo, the University of Cambridge data repository, at the following link https://doi.org/10.17863/CAM.116633 (42).

## Footnotes

a The mechanism for Korotkoff sound production remains widely debated (7). Common theories include turbulent flow due to the narrowing of the artery under cuff compression (8), and vibration of the artery walls and surrounding tissue upon opening of the artery (9, 10). While there is no conclusive theory to date, all proposed mechanisms require a change in cross-sectional area of the artery as described above (11), with the first opening of the artery corresponding to the first Korotkoff sound (12). In this work, we focus solely on artery reopening rather than the mechanism by which this opening results in Korotkoff sounds.

## Preprints

This manuscript was posted on a preprint: https://doi.org/10.48550/arXiv.2501.10232.
