# Supplementary material for: Underestimation of systolic pressure in cuff-based blood pressure measurement
Source: PNAS Nexus. 2025 Aug 12;4(8):pgaf222. doi: 10.1093/pnasnexus/pgaf222 (PMC12343083; doi:10.1093/pnasnexus/pgaf222)
Supplement: pgaf222_Supplementary_Data [file pgaf222_supplementary_data.pdf]

Supplementary Material for

Underestimation of systolic pressure in cuff-based blood pressure measurement

Kate Bassil and Anurag Agarwal

Kate Bassil
E-mail: kjb83@cantab.ac.uk

This PDF file includes:

Supplementary text
Figs. S1 to S5
Supplementary Material References

Supplementary Material Text

Using water as the working fluid

Recreating the non-Newtonian properties of blood is not essential to the investigation of auscultatory measurement, with results obtained using water and glycerol, and steer blood showing good agreement (1). Glycerol is sometimes used to increase the viscosity of water to provide a better match to blood (2). However, the mechanics of auscultatory measurement have been shown to work over a large range of viscosities (varying by a factor of 30), covering that of water and blood (2). While the exact measurements would be influenced by higher viscosity due to the increased resistance to flow, the general trend, lower downstream pressure resulting in greater underestimation, would be unaffected. We have therefore used water as the working fluid throughout our experiments.

A note on deflation rate

In our experimental rig, the deflation rate is influential because a high deflation rate results in a much larger drop in cuff pressure in the time taken for the artery to open. We demonstrate this by measuring the underestimation over a range of deflation rates, with a fixed downstream pressure of 20 mmHg. The results from this test are given in Fig. S4. There is a strong positive correlation between deflation rate and underestimation. In vivo, the time taken for the artery to open is short, of the order of 0.1 seconds (3), compared with the order of seconds in the rig. Therefore, with a recommended deflation rate of 2–3 mmHg, the cuff pressure would decrease by only 0.2–0.3 mmHg in the time taken for the artery to open. The error caused by the deflation that occurs while the artery is opening is, therefore, much smaller than the underestimation due to the low downstream pressure. Hence, in vivo, the deflation rate will not significantly affect the underestimation resulting from the low downstream pressure.

However, the deflation rate is still important in auscultatory measurement, as it determines how much the cuff pressure will have decreased from one systolic peak to the next. The resulting source of error is called cardiac cycle phase uncertainty and is explained in detail in (4). The key result is that cardiac cycle phase uncertainty will produce an average underestimation of systolic pressure of “one-half of the per-cardiac-cycle cuff deflation decrement”, and an equal overestimation of the diastolic pressure. With the recommended deflation rate of 2-3 mmHg, cardiac cycle phase uncertainty produces less than 2 mmHg underestimation of SBP (5), compared with an average underestimation of systolic pressure of 5.7 mmHg found in meta-analysis (6). Cardiac phase uncertainty is not significant enough to explain the observed level of systolic pressure underestimation, particularly when considering the known causes of overestimation counteracting the underestimation.

Cardiac cycle phase uncertainty is not relevant to our experimental results, where the upstream pressure is not oscillatory, and, as explained above, does not affect the physics described in the ‘Application to in vivo measurement’ section in the main text. It is included here for completeness and to avoid any uncertainty about the influence of deflation rate.

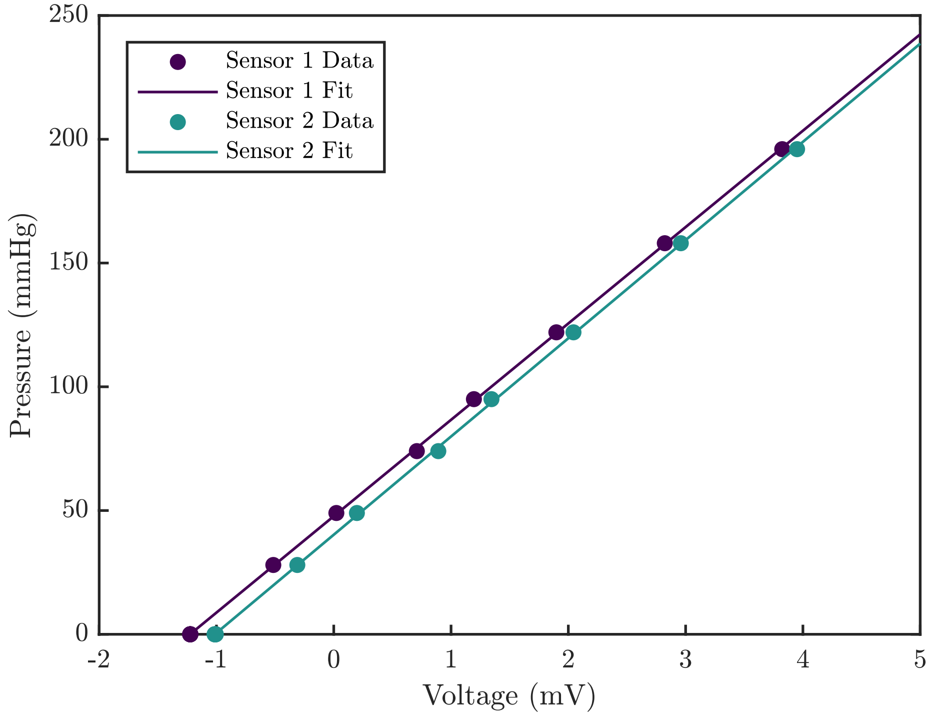

Fig. S1. Calibration plot for Honeywell sensors. The best fit calibration relationships are $P_1 = 38938.3 \times V_1 + 47.7$ and $P_2 = 39681.6 \times V_2 + 40.2$, where V_1 and V_2 are the voltages recorded from the sensors at positions 1 and 2 respectively in Fig. 3 in the main text, in V, and P_1 and P_2 are the pressures recorded on the mercury manometer, in mmHg. Both fits have an $R^2 > 0.999$.

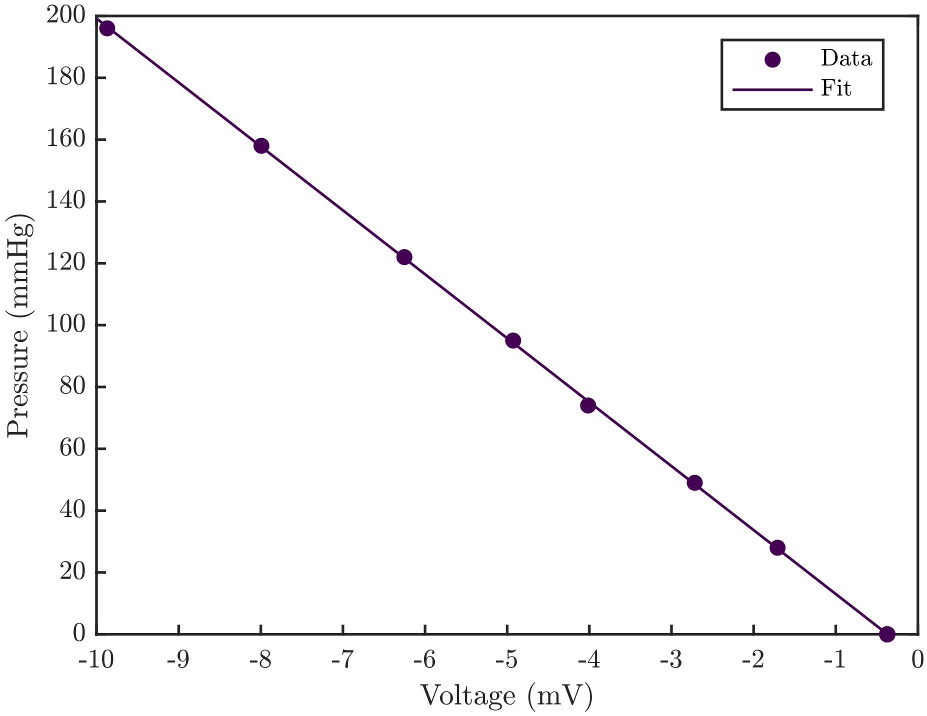

Fig. S2. Calibration plot for Kulite sensor measuring cuff pressure. The best fit calibration relationship is $P_{\mathrm{cuff}} = -20682.2 \times V_{\mathrm{cuff}} + -7.6$ where V_{cuff} is the voltage recorded from the Kulite sensor in V, and P_{cuff} is the pressure recorded on the mercury manometer, in mmHg. $R^2 > 0.999$.

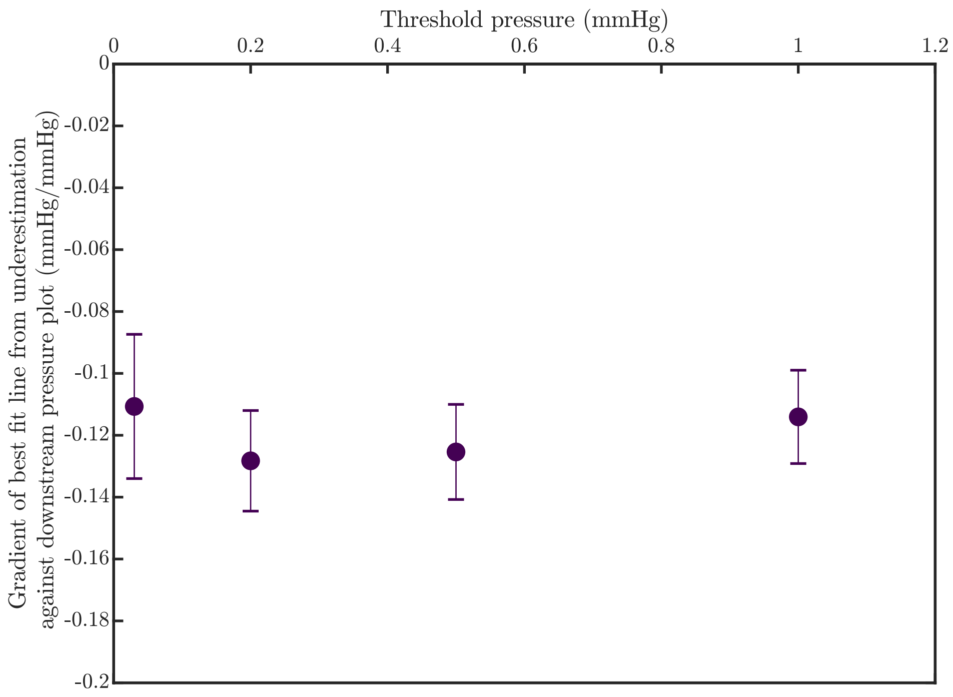

Fig. S3. The gradient of the underestimation-downstream pressure plots, as in the best fit line in Fig. 5B in the main text, for different threshold pressures. The threshold pressure is the rise in downstream pressure chosen to indicate the opening of the artery. Error bars indicate the 95% confidence interval for each gradient.

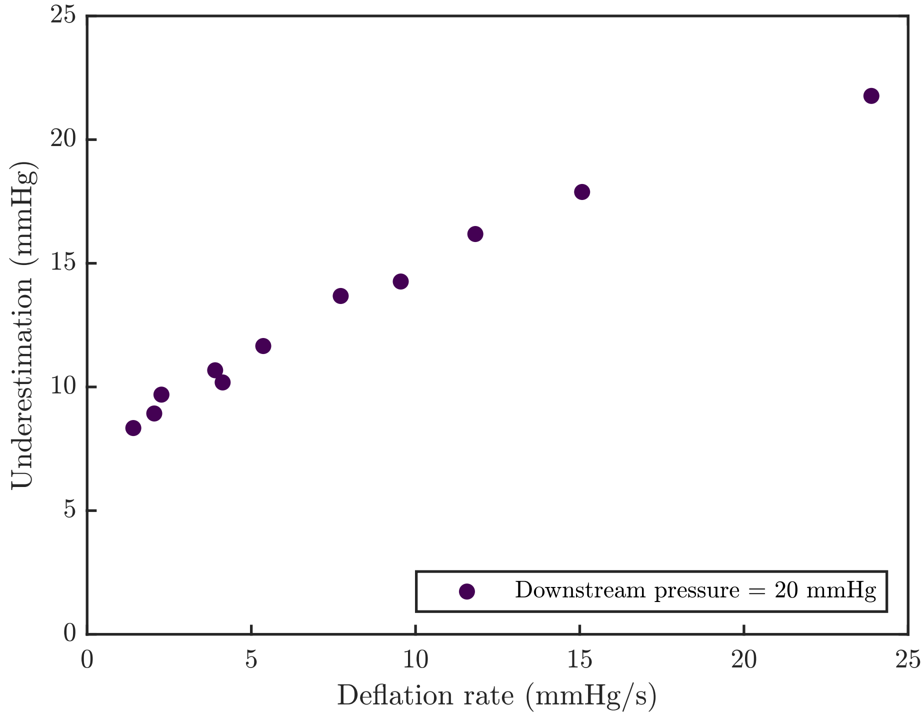

Fig. S4. Underestimation of upstream pressure against deflation rate. Tests were carried out using the pressure chamber to apply the external load, with a downstream pressure of 20 mmHg

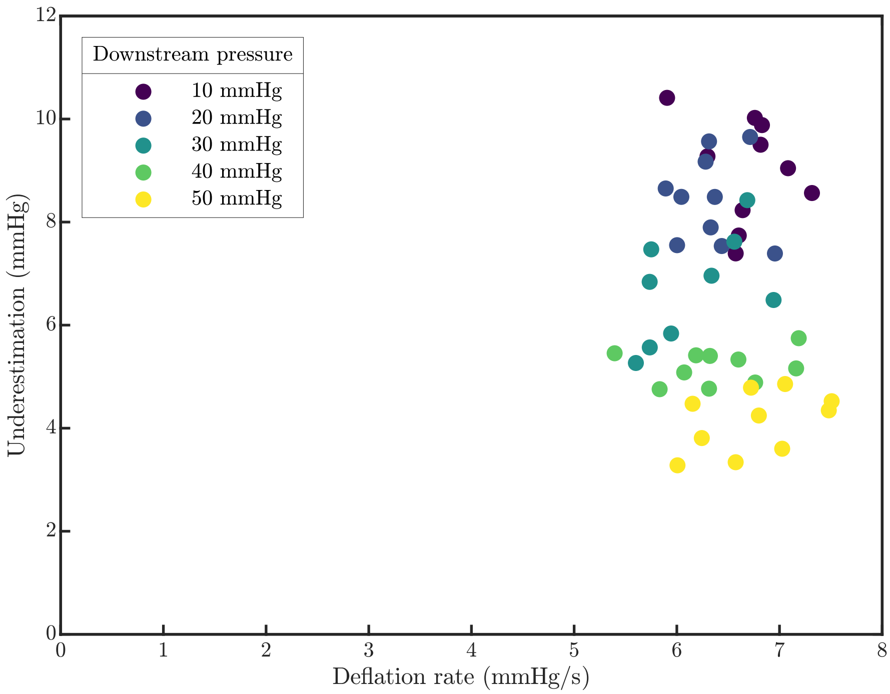

Fig. S5. Underestimation against deflation rate for the data plotted in Fig. 5B, over five downstream pressures, showing the narrow range of deflation rates over which these tests were carried out.

References

1. JA Burnell, KR Raman, AH Sacks, A study of auscultatory blood pressures in simulated arteries (https://ntrs.nasa.gov/citations/19670005250) (1965) accessed 16-08-2024.
2. M Anliker, KR Raman, Korotkoff sounds at diastole - a phenomenon of dynamic instability of fluid-filled shells. *Int. J. Solids Struct.* **2**, 467–491 (1966).
3. J Baranger, et al., The fundamental mechanisms of the Korotkoff sounds generation. *Sci. Adv.* **9**, eadi4252 (2023).
4. CP Hatsell, Cardiac cycle phase uncertainty: another source of error in indirect blood pressure measurement. *J. Med. Eng. & Technol.* **16**, 157–158 (1992).
5. D Zheng, JN Amoore, S Mieke, A Murray, How important is the recommended slow cuff pressure deflation rate for blood pressure measurement? *Annals Biomed. Eng.* **39**, 2584–2591 (2011).
6. DS Picone, et al., Accuracy of cuff-measured blood pressure: systematic reviews and meta-analyses. *J. Am. Coll. Cardiol.* **70**, 572–586 (2017).
